# Perspectives on Resuscitation Decisions at the Margin of Viability among Specialist Newborn Care Providers in Ghana and Ethiopia: A Qualitative Analysis

**DOI:** 10.1186/s12887-022-03146-z

**Published:** 2022-02-17

**Authors:** Sharla Rent, Ashura Bakari, Sara Aynalem Haimanot, Solomie Jebessa Deribessa, Gyikua Plange-Rhule, Yemah Bockarie, Cheryl A. Moyer, Stephanie K. Kukora

**Affiliations:** 1 Department of Pediatrics, Duke University, Durham, USA; 2 Suntreso Government Hospital, Ghana Health Service, Kumasi, Ghana; 3 Department of Pediatrics and Child Health, St. Paul’s Hospital Millennium Medical College, Swaziland St, Addis Ababa, Ethiopia; 4 Department of Pediatrics, Komfo Anokye Teaching Hospital Okomfo Anokye Road, Kumasi, Ghana; 5 Interberton Road, Cape Coast Teaching Hospital, Cape Coast, Ghana; 6 Departments of Learning Health Sciences and Obstetrics and Gynecology, Michigan Medicine, University of Michigan, Ann Arbor, USA; 7 Division of Neonatal Perinatal Medicine, Department of Pediatrics, Michigan Medicine, University of Michigan, Ann Arbor, USA

**Keywords:** Neonatal Resuscitation, Margin of Viability, Newborn Health, Global Health, Ghana, Ethiopia, Neonatal Ethics

## Abstract

**Background:**

In high income countries, guidelines exist recommending gestational age thresholds for offering and obligating neonatal resuscitation for extremely preterm infants. In low- and middle- income countries, this approach may be impractical due to limited/inconsistent resource availability and challenges in gestational dating. Scant literature exists on how clinicians in these settings conceptualize viability or make resuscitation decisions for premature infants.

**Methods:**

Qualitative interviews of interprofessional neonatal clinicians were conducted in Kumasi, Ghana, at Komfo Anokye Teaching Hospital and Suntreso Government Hospital, and in Addis Ababa, Ethiopia, at St. Paul’s Hospital Millennium Medical College. Transcribed interviews were coded through the constant comparative method.

**Results:**

Three discrete major themes were identified. The principal theme was a respect for all life, regardless of the likelihood for survival. This sense of duty arose from a duty to God, a duty to the patient, and a duty intrinsic to one’s role as a medical provider. The duty to resuscitate was balanced by the second major theme, an acceptance of futility for many premature infants. Lack of resources, inappropriate staffing, and historically high local neonatal mortality rates were often described. The third theme was a desire to meet global standards of newborn care, including having resources to adopt the 22–25-week thresholds used in high income countries and being able to consistently provide life-saving measures to premature infants.

**Conclusions:**

Neonatal clinicians in Ghana and Ethiopia described respect for all life and desire to meet global standards of newborn care, balanced with an awareness of futility based on local resource limitations. In both countries, clinicians highlighted how wide variations in regional survival outcomes limited their ability to rely on structured resuscitation guidelines based on gestational age and/or birthweight.

## Introduction

Preterm delivery remains a worldwide problem, with 10.6% of infants each year born prior to 37 weeks gestation and 4.1% of these preterm births occurring prior to 28 weeks [1]. For these extremely preterm infants, there is a significant gap in survival noted between high income countries (HICs) and low- and middle-income countries (LMICs), with over 90% of babies born before 28 weeks gestation surviving in HICs but only 10% of babies in this same gestational age range surviving in LMICs – a phenomenon known as the “90:10 survival gap” [2]. Despite its ongoing global prevalence and LMICs predominance, prematurity and ethical approaches to periviable delivery have largely been viewed through the lens of HIC health systems.

Advances in neonatal medical care over the past several decades have led to improvement in survival for infants delivered at extremely premature gestations in HICs [3–6]. Concurrent but more tempered progress has been seen in the risk of long-term morbidity and neurodevelopmental impairment among survivors [3, 7]. At this “margin of viability,” or the gestational age at which survival outside the womb is possible but unlikely, questions arise regarding for which infants intensive care is beneficial and for which it is not. In most HICs, the gestational age limits at which resuscitation is offered are often based on presumed physiologic limitations, epidemiologic outcomes, and local guidelines. Across HICs, prevailing this limit has been incrementally lowered to current thresholds in the range of 22 to 25 weeks [8, 9]. Differences in outcomes remain and derive from center size, experience managing premature infants, and institutional attitudes towards active provision of care at early gestational ages [9–11]. Guidelines from professional organizations discuss general situations in which it is appropriate to withhold resuscitative measures[12–15], but do not mandate specific thresholds for offering and obligating intensive care. Importantly, institution-specific guidelines are rarely published, and consensus is lacking on if guidelines should be established at the institutional, regional, or national level [16, 17]. Even in settings where guidelines are present, adherence to such guidelines varies [18, 19].

Generally, an approach that attempts to balance the different facets of the bioethical principles of beneficence and non-maleficence – namely providing treatment to those in need while avoiding futile treatment – is utilized across settings. When survival is physiologically impossible, the burdens of intensive care outweigh benefits, and resuscitation is ethically impermissible to avoid unnecessary harm to the infant and unfair use of resources. At gestations where there is a high probability of intact survival, resuscitation is obligated in the best interest of the infant. In the gestations between, a wide range of potential outcomes are possible, including disabling sequelae that may be viewed by some as unacceptable. For these patients, resuscitation is offered but not obligatory, and parental authority guides the decision whether to pursue intensive therapies. Due to the high degree of prognostic uncertainty for individual infants delivered at extremely preterm gestations, there remains continued debate regarding what the thresholds should be for offering and obligating resuscitation, and how these thresholds should be determined [20, 21].

A key challenge in any discussion of periviability is that newborn survival is extremely context dependent. In HICs there is a 50% chance of survival at 24 weeks gestation, whereas the same odds do not occur until 34 weeks in many LMICs [2]. Limitations in imaging and gestational dating further complicate prognostication based on gestational age in many LMIC settings, prompting many centers to use a weight cut-off for resuscitation rather than relying on gestational age. The relatively high prevalence of fetal growth restriction and small for gestational age (SGA) infants, however, present a challenge as prematurity is associated with a higher rate of neonatal death than SGA status [22]. Existing frameworks, formulated in HICs, focus on structed team-based decision making [23] and shared-decision making with families [24], but the existence or applicability of such frameworks to resource-constrained settings has not been well studied. Scant literature exists on how healthcare workers in LMICs conceptualize viability or make decisions at the time of delivery for very premature infants. To address this gap, we sought to explore the perspective of healthcare providers in two LMIC: Ethiopia and Ghana. Through an exploratory qualitative study, we investigated how specialist newborn care providers in higher level facilities within these two countries navigate these complex birth scenarios.

## Subject and methods

This qualitative study explored provider perceptions surrounding neonatal viability and resuscitation decision-making in Ethiopia and Ghana.

### Study context

Qualitative interviews took place in three hospitals in two major sub-Saharan African cities. Each medical center had an on-site physician serving as the local study lead who helped obtain IRB approval and briefed their respective Neonatal Intensive Care Units (NICUs) and Labor and Delivery (LD) wards about the study. Sites in Ethiopia and Ghana were chosen based on pre-established partnerships with the University of Michigan.

In Addis Ababa, Ethiopia, interviews were conducted at St. Paul’s Hospital Millennium Medical College (SPHMMC). SPHMMC is a teaching hospital that serves as a referral center for the large urban and rural region surrounding Addis Ababa. There are approximately 1000 deliveries and 200–300 admissions to the NICU per month. According to hospital records, mortality among preterm infants in this hospital is most often attributable to respiratory distress syndrome, sepsis, birth asphyxia, and congenital anomalies. At the time of the interviews, mechanical ventilation was not available in the NICU, and continuous positive airway pressure (CPAP) was administered through conventional bubble CPAP circuits. Dextrose and saline containing fluids were utilized for nutrition and hydration support for extremely premature infants. At times, the supply of nasal cannulas, pulse oximeters, and IV fluid catheters were fewer than the number of infants needing them. Multiple infants shared incubators to accommodate the high volume of patients, and patient to nurse ratios were as high as 10:1.

In Kumasi, Ghana, interviews were conducted at the Komfo Anokye Teaching Hospital (KATH) and at Suntreso Government Hospital (SGH). KATH serves as the main referral center of Kumasi, Ghana, in the heart of the Ashanti region which includes most of central and northern Ghana. It is the teaching hospital associated with the Kwame Nkrumah University of Science and Technology School of Medical Sciences. KATH is. KATH provides Level 3 care to 12,000 deliveries per year, as well as 400 Mother and Baby Unit admissions per month. At the time of the study, the hospital had two mechanical ventilators with CPAP capabilities; improvised CPAP using plastic water bottles was occasionally employed in cases of high patient volume. The hospital had surgical capacity, but did not have access to blended oxygen or parenteral nutrition. The leading causes of preterm mortality at KATH were infection and respiratory distress syndrome, according to mortality records. While the hospital was the best equipped in its district, demand for services often exceeded capacity for care.

SGH is a government-run district hospital with Level 2 newborn facilities in Kumasi, Ghana that refers its critical patients to KATH. It is located two kilometers from the main teaching and referral facility. SGH has approximately 250 deliveries and 120 Mother and Baby Unit admissions per month. During interviews, the Mother and Baby Unit relied on improvised CPAP units using plastic water bottles. The unit did not provide parenteral nutrition, but relied on dextrose and saline solutions for nutrition of infants unable to feed. Extreme preterm infants were also supported with administration of caffeine citrate. The hospital lacked blended oxygen and continuous monitoring equipment for preterm saturations.

Neither Ethiopia nor Ghana has official national, guidelines dictating resuscitation of extremely preterm infants. The included study sites did not have formal, institution-specific guidelines for resuscitation of extremely preterm infants at the time of this study.

### Research participants

A purposive sample of senior physicians, physicians in training, nurses and midwives at the participating study sites were interviewed, with specific participants selected based on availability during the interview period. Inclusion criteria were an ability to converse in English, with minimal translation assistance, and experience of at least 1 month working with newborn infants. All providers meeting these criteria who wished to partake in an interview were able to do so. One month of experience was chosen at the recommendation of local site leads, as this was felt to be the minimum amount of time necessary to be able to describe one’s beliefs in the context of hospital practices. The number of subjects was determined based on timeframe and a desire to obtain a diversity of views from different specialties until saturation of responses was reached. Saturation was determined when no new information was gained in subsequent semi-structured interviews.

### Data collection

Interviews at all sites were conducted in English by a trained neonatologist and qualitative researcher (SR). Verbal consent was obtained prior to each interview and participants had the ability to withdraw their consent at any time. Interviews were conducted privately or, in two cases, one in Ghana and one in Ethiopia, in groups of two at the interviewees’ request based on their personal comfort participating in an interview. At each site a private office was identified in which interviews took place. All were audio-recorded and transcribed verbatim. Data were collected for 2 weeks in each country. Interviews took place in Ethiopia in January 2018 and in Ghana in July–August 2018. Each interview session took approximately 30 min to complete.

Participants were asked a series of questions consisting of open-ended and short-answer responses, with follow-up probes when appropriate. To assess provider perceptions of viability, questions were asked regarding national, institutional, and personal guidelines for the resuscitation of very premature infants. Specific questions regarding a “cut-off” of 28 weeks were asked of each interviewee, with follow-up questions focused on their personal beliefs regarding this threshold. All interviewed healthcare providers were asked about the impact of resource-limitations on decision making in premature infants, as well as if they agreed or disagreed with how limited resources were allocated across gestational ages. If the healthcare provider raised the issue of religion or spiritual practices, further elaborative questions were posed to clarify the role of personal beliefs on clinical practices. Providers were not asked to disclose any religious affiliation.

### Analysis

Transcribed interviews were stripped of any identifiers and input into nVIVO 10.0. The study approach was guided by the consolidated criteria for reporting qualitative research (COREQ) [25] and interviews were coded through the constant comparative method of theme generation. The research team met regularly throughout the analysis phase to identify initial themes arising from health care provider responses. Initial coding was performed by SR and SK, with intercoder discrepancy discussed amongst the research team and resolved by consensus with CM determining final codes if disagreements remained. Team meetings focused on expanding or narrowing ambiguous codes, and creating additional codes when necessary, based on ongoing analysis. Grounded theory, an approach for collecting and analyzing qualitative data without imposing previously constructed theoretical frameworks [26, 27], was utilized to characterize healthcare provider perspectives without presuming that Ghanian and Ethiopian clinicians would conform to the researchers’ ideas about neonatal care and resuscitation decision making.

### Ethics

Ethical clearance was obtained from the institutional review boards at the University of Michigan (HUM00139420) St. Paul’s Hospital (REF: P.M. 23/164) and Komfo Anokye Teaching Hospital (REF: CHRPE/AP/193/18). Each participant was taken through a verbal consent process, which included explicit permission to audio-record the interview.

## Results

A total of 40 healthcare providers participated in the study. Of these, 20 were from SPHMMC, 11 from KATH, and 9 from Suntreso Hospital. Amongst providers, 3 of the physicians held hospital leadership roles and 5 had completed part of their training in another country (2 United Kingdom, 1 United States, 1 South Africa, 1 India). Additional breakdown of participants by role and site can be seen in Table 1

**Table 1:** Roles of Interviewed Medical Providers

		Partner Institution			
Role Grouping	Specific Role	SPHMMC	KATH	Suntreso Hospital	Total
Delivery Room Resuscitation Provider	ALS Team	3	NA	NA	7
	Midwifes	0	1	3	
Nurses	NICU Nurses	5	4	2	12
	Nursing Managers	1	0	0	
Mid- Level Care Providers	House Officers	0	1	1	12
	Pediatric Residents	7	1	1	
	Pediatric Physician Assistants	NA	NA	1	
Senior Level Physicians	Pediatricians	2	1	1	9
	Neonatology Fellows	1	NA	NA	
	Neonatologists	NA	3	NA	
	Other Physician	1	0	0	
Total Providers Interviewed		20	11	9	40

Three discrete overarching themes emerged related to viability, resuscitation, or intensive care provision for premature infants. The first prevailing theme was a respect for all life. This was balanced by the second theme, an awareness of futility. The third theme was a desire to meet global standards of newborn care. Each interviewee had responses in more than one of the themes and no one declined to answer any of the questions posed.

### Respect for all life: *“because they are alive, we resuscitate them.”*

The principal theme was a respect for all life, regardless of the likelihood for survival. Nearly every provider said that they would resuscitate any infant “born with signs of life”, commenting both on the difficulty of deciding whether another person should live or die, and on their personal duty to preserve life whenever possible. The sense of duty described in the interviews comprised a duty to God, a duty to the patient, and a duty intrinsic to one’s role as a medical provider. Those citing religious beliefs as the driving force for universal resuscitation attempts indicated that it is up to God to decide if a baby lives or dies, not the medical providers themselves. Several providers noted that life begins at conception, and this requires them to intervene on any live-born infant. Representative quotes on this topic are shown in Table 2**.**

**Table 2:** Provider Quotes Addressing Duty

Provider Quotes	Perceived Duty
*“If the doctor believes that God created the life or that God gave the woman the pregnancy, if the doctor believes that then he should also believe that God can do a miracle and the baby can survive. So, there is no harm in trying.”* ~ *Mid-Level Provider, Ghana*	To God
*“There is life in the baby, so I have to do my part. If the baby dies, it is not me. If I leave it [and do not provide resuscitation] and the baby dies, God will ask me. I didn’t swear for that. In my training I said, ‘I will help you’.”* ~ *Delivery Room Provider, Ghana*	To God To the Patient
*“We don’t think that they will actually survive. It is more that ethically you can’t leave them there.”* ~ *Mid-Level Provider, Ethiopia*	As a Medical Provider To the Patient
*“Once there is life … once the baby is born with life, it is a baby. It is a human being. We have to resuscitate and take good care of the baby. It is our responsibility.”* ~ *Mid-Level Provider, Ghana*	As a Medical Provider To the Patient
*“When the baby comes out, if he is alive, if we see some sign of life, then we just resuscitate. We don’t even ask the mother. We resuscitate them and we keep them in the NICU.”* ~ *Senior Physician, Ethiopia*	To the Patient

In Ethiopia, several providers volunteered their stance against abortion, based on religious and personal beliefs, as additional reasoning for why they would never withhold resuscitation. Providers in both countries shared that even in circumstances of a medically induced abortion, if the infant is born with signs of life, they then have a duty to resuscitate. Provider tone when explaining this concept ranged from matter of fact to frustrated, with some providers expressing that it was unreasonable to expect them to resuscitate these extremely preterm babies. Regardless of their personal beliefs, all interviewees felt challenged by having to manage these situations.

*“If the baby has life, some kind of movement, then we resuscitate. Even if the mother doesn’t want us to … most of the time the mother has had some kind of complication and is has been told to her that she is going to have a medical abortion. Misoprostol will be given to her, but the baby will be born alive … When the baby comes out, if he is alive, if we see some sign of life, then we just resuscitate. We don’t even ask the mother. We resuscitate them, and we keep them in the NICU.”* ~ *Senior Level Physician, Ethiopia*

*“How can somebody abort at 5 months and then you ask me to make the baby survive? Am I a magician?” ~ Delivery Room Provider, Ghana*

Several providers explained how their duty to resuscitate was driven by uncertain outcomes. Because it was theoretically possible for a very premature infant to survive, they felt a responsibility to try even if the odds of survival were low. Most physicians and nurses could think of at least one infant who survived under 1000 g or before 28 weeks, and this gave them hope that other very premature infants could survive as well.

*Provider: “Usually they will not be salvaged, but even if we know the prognosis is very poor, we will support them as we can. Our feeling is that the prognosis is very poor.”*

*Interviewer: So why try?*

*Provider: Yah, we have to try. They might be salvaged. Sometimes. We had one baby, whose birthweight was like 700g, who has grown up here. Sometimes, if we are lucky enough, we might be able to save that one.”* ~ *Mid-Level Provider, Ethiopia*

### Awareness of futility: *“after 2 or 3 days they will be too complicated and deteriorate”*

This widely held sense of duty to resuscitate any infant born with signs of life was balanced by an acceptance of futility in many cases of premature infants. Providers cited a lack of resources, inappropriate staffing, and historically high local neonatal mortality rates. A combination of several factors was thought to be contributing to the high mortality in premature infants, and senior physicians detailed how a multi-pronged approach would be needed before a change in outcomes would be seen.

*Those above 30, 32 weeks usually recover. Those less than 30 weeks, especially those less than 28 weeks, they stay maybe 4 days. The typical is 4 days of life, no more than a week. The outcome is no good. First, I think we need to improve our care and improve our outcomes before we would modify this definition. Otherwise, the outcome will not change.* ~ *Senior Physician, Ghana*

Physicians also commented on institutional practices of restricting what resources are used on premature infants, electing to allocate their limited resources towards newborns with a higher chance of survival.

*“Anyone who comes out crying or anything, they will get a chance. Of course, the smaller you are, the less aggressive the chance is.” ~ Senior Physician, Ghana*

*“Since we don’t give them any treatment to mature the lungs, they die from their respiratory system.” ~ Nurse, Ethiopia*

*“If you had less than 26 weeks, it is unlikely that we would go all out with bubble CPAP and everything. We do have a local improvised CPAP, so it is likely that that is the highest that we would do for that baby.”* ~ *Senior Physician, Ghana.*

The prevailing attitude at all 3 centers was that while it was unacceptable to deny the patient resuscitative efforts at the time of delivery, it was equally inappropriate to expend significant effort and resources on infants who were unlikely to survive. In Ethiopia, the highest level of support offered was an improvised CPAP machine made from compressed air tanks and water bottles. In Ghana, the academic NICU had two ventilators, but these were reserved for term newborns. A premature infant at this institution could be placed on traditional CPAP. At the district hospital, compressed air was available but most premature infants were transferred to the academic center for further management. IV fluids were available at all centers, as were antibiotics. Several clinicians cited the lack of total parenteral nutrition and surfactant as reasons why premature infants did not survive. The most reported causes of death in these infants were sepsis, respiratory distress, and asphyxia.

When asked about any long-term complications of prematurity that may impact decision making either at resuscitation or in the NICU, a few providers commented that developmental delays can occur in preterm or asphyxiated newborns. Such infants, and their families, were also at risk of being ostracized due to a misattribution of the infant’s condition to a curse or sin. Providers went on to explain that many of these children are kept at home, inside, at all times and eventually pass away due to complications or neglect.

*“The consideration is, if we resuscitate, they might end up with disabilities like cerebral palsy and developmental delay. This is very difficult for the families, to accept that. There are patients who are born premature and who are disabled. They cannot take care of themselves after growing. They cannot eat by themselves. They cannot analyze things … There is also cultural stigma. Maybe, this preterm baby born and having cerebral palsy, the society considers that some sort of sin. Because his family or her family is doing something bad, so that is why God will give them a baby like this. This is what people believe to be true.” Mid-Level Provider, Ethiopia*

Most providers within our study did not identify any additional potential down-sides to resuscitation of incredibly preterm infants. Only providers who had trained abroad commented common morbidities seen in surviving premature infants in HICs, such as bronchopulmonary dysplasia or retinopathy of prematurity, noting that any infant sick enough to develop those conditions would not survive in their set-up. Similarly, no provider within our study commented on the potential for “suffering” due to burdensome interventions or a prolonged NICU stay.

### Desire to meet global standards: *“so sometimes we feel like, ok, if i do it, maybe it will work. and maybe that is why we don’t give up”*

Despite the variation amongst providers regarding specific resuscitation practices for preterm infants, most expressed a desire to work towards meeting “global standards of care”. For some, this meant having appropriate resources to be able to directly adopt the 22–25-week cut-offs used in the US. For others, it meant providing life-saving measures to any child that needed it.

*“I think, maybe, since we see that there are some countries that are able to salvage those babies that are 20 weeks, even some that are 18 weeks, I am not sure, but 20 weeks. But even if the baby is 18 weeks, 16 weeks, and has signs of life, then we have to try and save him. Whether he can be saved or not, that is a different matter. For us, we have to try and save that baby, if it is possible.”* ~ *Senior Physician, Ethiopia*

Many felt that having guidelines that mirrored those from more developed nations was necessary to be taken-seriously on the international stage. Similarly, many felt updated regulations were likely forthcoming as survival continued to improve at lower gestational ages. Discussing this issue, providers frequently used the term “abortus”, to refer to a miscarriage or early stillbirth (before 28 weeks) rather than a late stillbirth (after 28 weeks).

*“Why is our definition for abortion different [than the definition in America]? It is better to deal with the ministry of health … they should deal with that.” ~ Mid-Level Provider, Ethiopia*

*“In Ghana, any baby less than 28 weeks is termed an abortus. So, maybe, with time, I am sure they are going to try and modify it. Because, now, we are having a lot of premature babies below 28 weeks here. At first, at 28 weeks, at 29 weeks, they were not surviving. Now, even 24 weeks are surviving. I am sure they will try and change that theory and maybe bring it to 24 weeks.” Mid-Level Provider, Ghana*

The majority of physicians and hospital leaders recognized the variation in resources within their own countries, which impact the ability to provide care at standardized levels. Both SPHMMC and KATH are regional referral centers, and physicians at these two centers were conscious of the relative advantage that afforded them in providing quality neonatal care.

*“Well, St Paul’s is a tertiary hospital. We have more capabilities than most centers. If you go to the district, the situation is different. 30 weeks, 32 weeks, they are dying. Mortality is very high. I don’t think 28 or 29 weeks could survive.” ~ Mid-Level Provider, Ethiopia*

*“We know that in our setting, if a baby has not completed all of these weeks, we try to do our best. We try our best to have the babies survive. What we know is that the survival rate does depend on our management and what we have available here … The ones we term an abortus also come with a lot of complications, complications that take them out. In our setting it is very difficult for such a baby to survive.” ~ Mid-Level Provider, Ghana (Suntreso Hospital)*

### Addressing a 28-week threshold: *“It is difficult to decide on a person’s life.”*

These three themes intersected for many providers when they were asked directly about the commonly referenced viability cut-off of 28 weeks. Nearly all provides felt that infants below 28 weeks were unlikely to survive in most centers in Ghana or Ethiopia. Providers were split on whether a cut-off of 28 weeks should be used, either institutionally or nationally, to inform resuscitation decisions. Most felt that the presence or absence of a guideline was irrelevant, both because near universal resuscitation attempts were already happening and because of the high mortality rate for these infants regardless of interventions. Representative quotes expressing views on both sides are shown in Table 3**.**

**Table 3:** Provider Quotes Addressing a 28 Week Cut-off for Resuscitation

Providers who Felt that the Cut-off should be at < 28 weeks or Nonexistent	Providers who Felt that the Cut-off should Remain at 28 weeks
*“28 weeks is just a number for us. If the baby is alive, if it is breathing, if he is doing well, we can transfer to the NICU … For each baby we must be flexible. We cannot see a baby less than 28 weeks and say, ‘he can die, she can die, I won’t help him’, that is not good.”* ~ *Delivery Room Provider, Ethiopia*	*“I agree with the law at 28 weeks. The survival is better at 28 weeks and above. Even if we resuscitate them younger, they don’t survive. They cannot.”* ~ *Nurse, Ethiopia*
*“Once a baby shows signs of life, we don’t follow the law. If the baby is showing signs of life, if the baby is here, what else can we do? … We don’t say if you are less than 28 weeks that we will not resuscitate you.”* ~ *Senior Physician, Ghana*	*“Considering the fact that we don't have a lot of stuff for advanced care, I would say that we keep it [the viability cut-off] at 28 weeks. If we manage to solidify our basics, then possibly we can move forward.”* ~ *Senior Physician, Ghana*
*“It is just decided that abortion is below the gestational age of 28 weeks and the birth weight of 1000 g. That is just a legal issue. Otherwise, we are still trying to save them as much as possible. Many babies with a birth weight of around 900 g have been saved here.”* ~ *Mid-Level Provider, Ethiopia*	

Emphasizing the argument that guidelines, even if present, would not drive clinical decision making for periviable infants, many physicians affirmed that resuscitation attempts are routinely being made before 28 weeks gestation, despite a prevailing belief that this is officially considered below the threshold for viability.

*But you cannot give numbers for clinicians. Numbers for a technician … sure they might use it … but any baby that is alive deserves resuscitation.” ~ Senior Physician, Ethiopia*

A few providers cited “ethics” in their rationale for why practice was not following policy.

*Provider: We resuscitate every baby, be it 23 weeks, 24 weeks, and so on. Now the problem is that because of our set-up, they may not have a good outcome. But we will resuscitate all of them. Every baby. From our point of view, and also from the ethical point of view, that should be the case.*

*Interviewer: Tell me more about that … the ethical point of view. How do you go about thinking about that?*

*Provider: You know, for us, every baby born breathing, with signs of life, we call it a live baby. Even if it is a preemie baby, still it is alive. We don’t consider them an aborted baby. We don’t. In our day-to-day clinical practice … now we don’t have a written guideline … but all of us practicing here, we don’t consider them as an abortus. We try to preserve their life as much as possible. ~ Senior Physician, Ethiopia*

## Discussion

To our knowledge, this is the first study of LMICs to evaluate provider perceptions of margins of viability and decision-making frameworks for preterm infants in these environments. Providers within our study balanced a respect for all life, regardless of gestational age, and a desire to meet global standards of newborn care with an awareness of futility based on local resource limitations. In both Ethiopia and Ghana, interviewed clinicians highlighted how wide variations in regional survival outcomes limited their ability to rely on structured resuscitation guidelines based on gestational age and/or birthweight, as is done in HICs. Prevailingly, those interviewed described making clinical decisions around resuscitation and viability following a dominant ethical framework centered on the intrinsic desire to preserve human life. This moral obligation to save lives was expressed both by those with high decision-making power within the health system, such as physicians, as well as by nurses and midwives. This approach developed in the context of low rates of survival below 28 weeks gestation and, consequently, limited societal and familial burden of long-term complications of prematurity. Our work builds upon the body of HIC literature addressing the ethical considerations for periviable care and upon the numerous global works highlighting the high neonatal and perinatal mortality rates in LMICs.

A near universal finding in our study was a desire to preserve life at any cost, reflected in a willingness to offer resuscitation to newborns regardless of gestational age. In line with the bioethical principal of justice, several providers felt that if available interventions were being used to save some babies, they were obligated to use these same interventions to try and save all infants. A few physicians, who had completed part of their training in high-resource centers abroad, pointed out, the “harm” of resuscitating at very low gestational ages does not yet exist in LMICs in the same way it does in HICs. Infants at these gestations are not intubated and do not receive extensive intensive care. Infants who survive at very premature gestations do so despite limited support; those who do not generally succumb to their prematurity within hours to days. As seen in provider comments related to futility, most premature infants do not survive, and providers are keenly aware of this. As many of the long-term morbidities arising from premature delivery in HICs are caused by the medical technology required to support these infants, such as mechanical ventilation related pulmonary injury resulting in BPD [28], premature infants born in LMICs who require this level of support are unlikely to survive the NICU and at low risk of these life-long complications. In contrast to many HICs, where parents’ values are regarded as a primary influence on decisions at the margin of viability [29], parental authority was not described as a major factor in resuscitation decision making by providers in our study.

While religious beliefs drove many providers in our study to advocate for universal resuscitation, many also pointed out how frequently infants with disabilities are viewed as a “sin” or a “punishment from God”. Providers, therefore, considered the potential familial burden and societal stigma when managing extremely preterm infants while simultaneously acknowledging the overall low rate of long-term developmental disabilities within their patient populations. Interviewed providers experienced with such “complications of prematurity” emphasized how, until overall survival rates improved, the emphasis would likely remain on overall survival rather than on considerations of disability and quality of life.

In describing their working definition for a “margin of viability,” many providers in our study referenced the commonly used definition of “late stillbirth” definition – fetal deaths at ≥ 1000 g or ≥ 28 weeks of completed gestation[2]—developed for international comparisons of pregnancy outcomes. Many providers felt that this definition, with an intended purpose of standardizing data collection, was being inappropriately extended into a clinical practice recommendation. Many interviewed providers cited 28-weeks as the “official” gestational age below which any infant “should” be considered a stillbirth or miscarriage, despite simultaneously disregarding this threshold in practice. This opinion is supported by other LMIC work, such as a study from Nigeria identifying 26 weeks gestation as the point where survival is expected to be greater than 50% [30].

Several clinicians mentioned the stark variation in survival in rural health centers compared to urban clinics, inconsistent supply chains, and limited resources to care for preterm infants as additional challenges in setting national resuscitation guidelines. The challenge of rationing care due to limited resources, and a lack of guidance on how to handle resource shortages in an ICU setting, has been previously described in the literature [31–33]. Compounding resource shortages is the relatively high cost of care for a NICU admission, despite proactive efforts by physicians to minimize direct costs to patients [34]. Highlighting the impact of limited resources within a NICU, providers at all centers noted that after surviving the initial resuscitation, the most premature infants would be allocated the fewest resources in the NICU. Almost paradoxically, the initial approach prioritizing efforts to save all infants in the delivery room shifts to a more utilitarian-focused framework once an infant has entered the larger NICU patient community, where scarce resources are allocated towards those most likely to survive. This practice mirrors findings of previous researchers who have commented on how conditions in resource-constrained settings often mandate resource allocation to be an integral component to NICU care [31, 35].

**Fig. 1:**
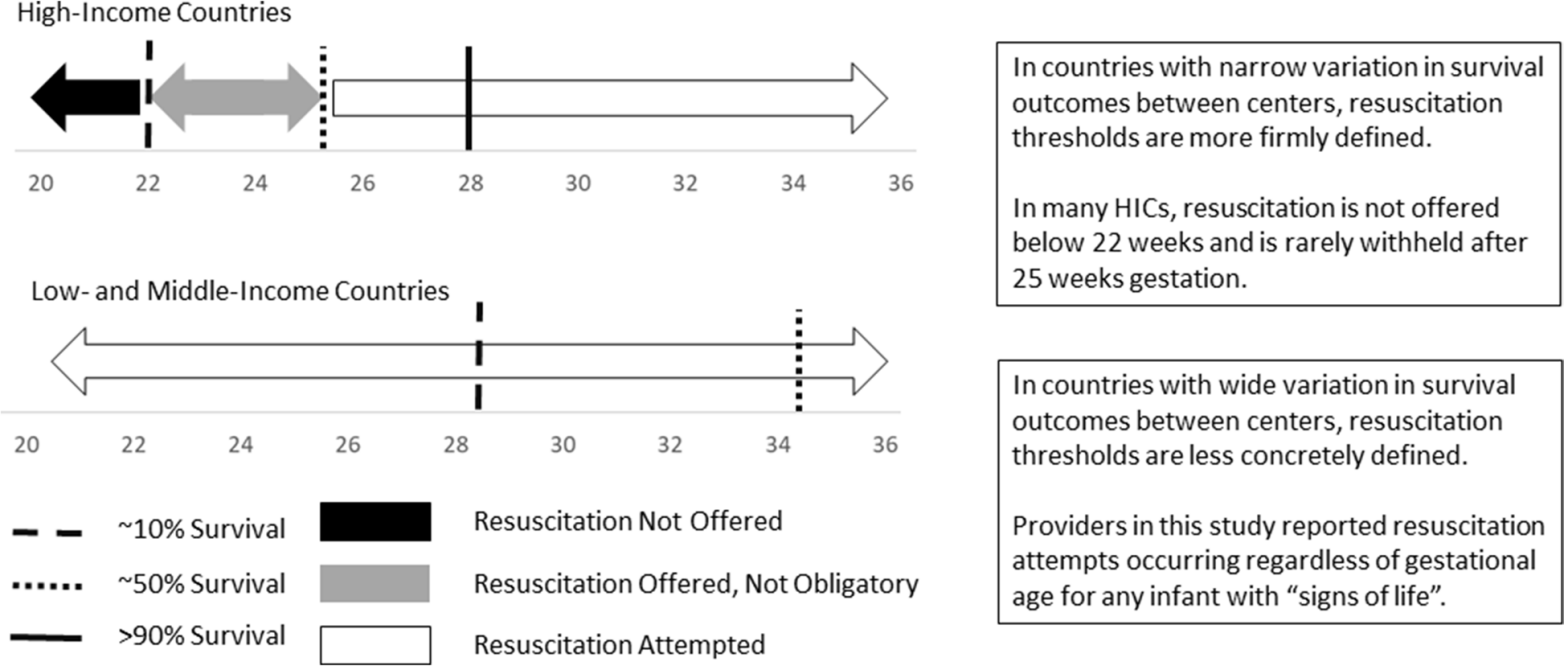
Influence of Survival Trends by Gestational Age on Resuscitation Practices

Likely one set of guidelines cannot be universally applied in the context of a high degree of variation in resources, access, and outcomes. This concept is detailed in Fig. 1. While in many HICs, there is relative uniformity in recommending resuscitation above 25 weeks and comfort care at or below 22 weeks gestation, variation in practice exists between these thresholds [36]. Globally, national guidelines draw from local outcome data [36], allowing them to be reflective of that country’s population. As the survival of premature infants continues to improve in countries such as Ethiopia and Ghana, clinicians will need to consider how to balance infants’ best interests against potential harms, weigh the potential of survival with significant morbidity against the risk of mortality, and engage with parents about their values regarding what potential quality of life outcomes are acceptable. The narrow guidelines for resuscitation at the margin of viability guidelines used in HICs were designed for clinical settings with reliable access to medical technology and supplies, and may not be feasible or appropriate in resource-constrained settings. Likewise, the ethical framework guiding periviable resuscitation decisions in the US and Europe developed in parallel to the advancing lifesaving technology, and were influenced by iterative evaluation of cultural and societal values about preserving life, avoiding harm, and allowing parental authority [21]. It is inappropriate to assume that healthcare professionals and parents in LMICs should adhere to ethical constructs developed within a culture distinct from their own. Just as there is some latitude in guidelines in within and between HICs based on local outcome data and cultural values, LMICs will need to develop their own frameworks for approaching resuscitation decisions for extremely premature infants in a way that balances best interests against non-maleficence, and encourages justice. These guidelines, like those in HICs, should not be static, but rather will need to undergo iterative change, as survival improves and resources become more available.

While it has been reported that in low-income settings preventable deaths are accepted as inevitable by parents and healthcare workers [2], our findings highlight the concomitant belief in a universal right to life and duty to actively provide of care. How these beliefs will intersect with the realities of managing patients with prolonged medical resource needs and severe morbidities as survival outcomes improve remains to be seen. While standardized approaches exist for preparing expectant parents and the healthcare team for delivery of a marginally viable infant [38, 39], the feasibility and acceptability of these approaches in LMICs is yet unknown. Likely, providers in LMICs, such as Ethiopia and Ghana, will develop effective local strategies to address complex management decisions regarding resource allocation and navigate increasing morbidity in the setting of improving mortality for premature infants.

### Limitations

This study has several limitations. First, for logistical reasons, a purposive sample of providers was used for the interview process. While this sample included individuals from many different medical roles, it is likely that their views do not represent those of all healthcare providers. The choice of two sets of two interviewees to interview together may also have biased those responses. Second, all interviews took place in major cities with tertiary care centers. While the results may reflect views of providers in similar health settings with high rates of preterm delivery and perinatal mortality, they are less likely to represent views of providers in rural areas with even further limited access to resources. Similarly, the sample size at each site of this study was relatively small and limited to 3 institutions, so the results should be interpreted accordingly. The inclusion criteria of being comfortable conversing in English also excluded some nurses and midwives, whose perspectives may have differed from the staff who were interviewed. While English language is used in medical training, the daily conversations at all hospital sites occurred in local languages. It is possible that these clinicians could be more likely to hold traditional views that infant conditions arise from curses or sins and thus possibly discourage resuscitation at early gestational ages. The use of English was not a limiting factor for physicians. Finally, some interviewees may not have felt comfortable expressing controversial views or may have withheld information from people seen as outsiders, potentially tailoring their responses to align with what they believed the visiting neonatologist desired or expected.

Despite these limitations, this study had many strengths. Our goal was to determine the current practice for defining the margin of viability in the included medical centers and examine provider viewpoints around dictating care for periviable infants. Through the shared input of 40 providers, we were able to describe a common management approach in this population, which was relatively uniform between sites, and explore the array of beliefs that inform these practice patterns. Relating identified themes to published and established ethical frameworks extends the value of this study.

## Conclusion

Medical providers in three NICUs in Ethiopia and Ghana expressed how local resuscitation practices are driven by a respect for all life and by a balanced awareness of futility based on local survival data. Interviewed providers overwhelmingly expressed that resuscitation below 28 weeks gestation was warranted and expected, as survival was possible at these gestations. This perspective must be interpreted within a context of very unlikely neonatal survival at these very preterm gestational ages and allocation of scarce resources away from very preterm infants within the NICU. Ultimately, the balance that LMIC healthcare providers will strike between preserving life at all costs and avoiding unnecessary suffering on the part of the patient has yet to be determined. Guidelines addressing viability and resuscitation decision making for preterm infants must not only derive from epidemiologic outcomes, but also acknowledge local variations in access to care, reflect prevailing cultural beliefs, and be adaptive to future advances in neonatal care. While the global neonatal community and bioethicists can provide experiential guidance and support in developing resuscitation frameworks in LMICs, the process should be driven by local health leaders. Further work is necessary to ascertain and incorporate the viewpoints of rural providers, families, and obstetric providers when developing viability guidelines.

## Data Availability

The datasets generated and/or analyzed in the study are not publicly available due as they contain information that could compromise the privacy and anonymity of research participants. Deidentified data are available from the corresponding author on reasonable request.

## Abbreviations

HICs: High income countries
LMICs: Low- and middle-income countries
SGA: Small for gestational age
NICU: Neonatal Intensive Care Unit
LD: Labor and Delivery
CPAP: Continuous positive airway pressure
SPHMMC: St. Paul’s Hospital Millennium Medical College
KATH: Komfo Anokye Teaching Hospital
SGH: Suntreso Government Hospital
COREQ: Consolidated criteria for reporting qualitative research
WHO: World Health Organization
